# Compliance With the Royal College of Radiologists Guideline for Actionable Reporting and Its Impact on Patient Care: A Retrospective Analysis of Reporting Practices From a Major Trauma Center

**DOI:** 10.7759/cureus.35921

**Published:** 2023-03-09

**Authors:** Utkarsh Sharma, Austin R Gomindes, Kritika Sharma, Jamaal Choudhry, Henry K C. Searle

**Affiliations:** 1 Major Trauma Services, Queen Elizabeth Hospital Birmingham, Birmingham, GBR; 2 Higher Education Academy, Advanced Higher Education - UK Professional Standards Framework, Birmingham, GBR; 3 School of Medical and Dental Science, University of Birmingham, Birmingham, GBR; 4 Trauma and Orthopaedics, University Hospitals of Coventry and Warwickshire, Coventry, GBR; 5 Paediatrics and Child Health, Lok Nayak Jai Prakash Narayan Hospital, New Delhi, IND; 6 Warwick Clinical Trials Unit, Clinical Sciences and Research Laboratories, University Hospitals of Coventry and Warwickshire, Coventry, GBR

**Keywords:** ct scan, reporting quality, reporting system, quality improvement research, royal college of radiology

## Abstract

Introduction

Prompt diagnosis forms the mainstay of management of any patient arriving at the hospital. In developed settings, apart from clinical assessment, imaging in the form of computed tomography (CT) scan plays a vital role in arriving at the patient diagnosis. The reporting should follow pre-defined Royal College of Radiologists (RCR) standards to improve the quality of the diagnostic process.

Objectives

To identify the compliance of reporting as per the RCR standards for the communication of radiological reports and fail-safe alert notification.

Materials and methods

A retrospective review of body CT scans was done in two cycles within a span of three months. A total of 100 randomized scans were assessed in each cycle, both from the A&E (accident and emergency) and inpatients. Normal scans and outpatient scans were excluded from the study. Data were collected using the online portal (CRIS) and statistical analysis was performed.

Results

After the first cycle of the audit, 95 reports out of 100 met the standard RCR criteria. After the second cycle, 97 reports met the criteria of the audit. One inpatient scan and two A&E reports did not meet the specified criteria in the second cycle.

Conclusion

After the two cycles of the audit carried out over three months, we were able to achieve almost 97% of reporting standards as compared to 95% obtained previously through a quality improvement project and create awareness.

## Introduction

The evaluation of critically injured patients with multiple trauma poses a significant challenge for healthcare providers in a Level 1 Major Trauma Center. The prompt and accurate diagnosis in such cases is crucial for the patient outcome, as the time taken in diagnosing the injuries has a direct impact on morbidity and mortality. Clinical assessment and imaging, particularly computed tomography (CT) scans, play a critical role in the management of polytrauma patients; in these cases, CT scans are widely used in developed settings for the rapid and accurate diagnosis of major injuries. It is the most sensitive and specific non-invasive diagnostic tool, saving time and providing accurate results. However, the quality of the diagnostic process also depends on the reporting of CT scans. A systematic and standardized reporting approach is necessary to ensure the maximum benefits of the CT scan are realized [1,2].

The Royal College of Radiologists (RCR) provides guidelines for the reporting of CT scans in polytrauma cases. The standard CT scan report should include patient details, clinical information, and the results of any prior investigations, both radiological and non-radiological. The report should also include the proposed management plan for the patient. The reporting should be systematic and standardized, following predefined standards set by the RCR [1-4].

The journey of an actionable report starts at the grassroots of its initial request from the request of appropriate imaging investigation for the appropriate patient and the appropriate time and finishes when a structured report with the use of standardized language and with the application of evidence-based standardized guideline, is returned to the requesting clinician. And hence radiologists would benefit from rethinking their working process with IT (Information Technology) integration and support from computing tools and potentially AI (Artificial Intelligence) in the future to be able to achieve these high standards to have an impact on patient care [5].

Objectives of the study

To identify the compliance of reporting as per the RCR standards for the communication of radiological reports and fail-safe alert notification.

## Materials and methods

Type of study

In the first cycle, 373 CTs were reviewed and excluded normal CT scans, CT head scans, CT intracranial angiograms, and outpatient CT scans to complete a total of 100 CT scans for the first cycle and during the second cycle a total of 420 CT scans were assessed and similarly excluded normal CT scans, CT head scans, CT intracranial angiograms, and outpatient CT scans and to complete a total of 100 CT scans to be used in the study.

The data collection was performed through the use of the Central Registration and Identification System (CRIS) (Aldershot, United Kingdom), and the RCR “Learning from discrepancies template” was used to document the findings of the review and at the same time provide feedback to the reporters (Figure 3 in Appendices) [2].

The reports were assessed and compared to the RCR reporting standards, independently by two authors (US and AG). Both authors reviewed the RCR reporting standards, and use them in common practice. Any conflicts were then discussed with a third party.

The RCR reporting standards outline the objectives of providing actionable reporting, answering the clinical question, and delivering a definitive or differential diagnosis in the presence of abnormalities (Table 1). The RCR standards for actionable reporting also emphasize the importance of providing appropriate advice for the next step of management, aligned with the patient's best interests [1-4].

**Table 1:** Reporting standards as per the Royal College of Radiologists The guidelines illustrating the core requirements to fulfill the criteria for the Royal College of Radiologists standards for actionable reporting.

Every department should aim to deliver actionable reporting
Every report should answer the clinical question
When an abnormality is described, the report should include definitive or differential diagnosis
Appropriate advice should be given for next step of management in patient’s best interest

Reports were collated for the first cycle. Then a presentation was made to the department on the importance of adherence to RCR. Then second-cycle data collection was reported in response to the findings of the first audit cycle, a departmental presentation was made to emphasize the importance of reporting according to the RCR guidelines. The presentation was also circulated to individuals who were unable to attend in person.

Data analysis

Data will be reported as raw percentages for adherence to RCR standards (yes versus no). Statistical analysis was performed using Statistical Product and Service Solutions (SPSS) (IBM SPSS Statistics for Windows, Version 29.0, Armonk, NY). A Chi-squared test was performed to compare differences between adherence to the RCR reporting standards (yes versus no) before and after the presentation. A p-value of <0.05 will be determined as significant.

## Results

There were no conflicts between the two authors who reviewed adherence to the reporting standards.

**Figure 1:**
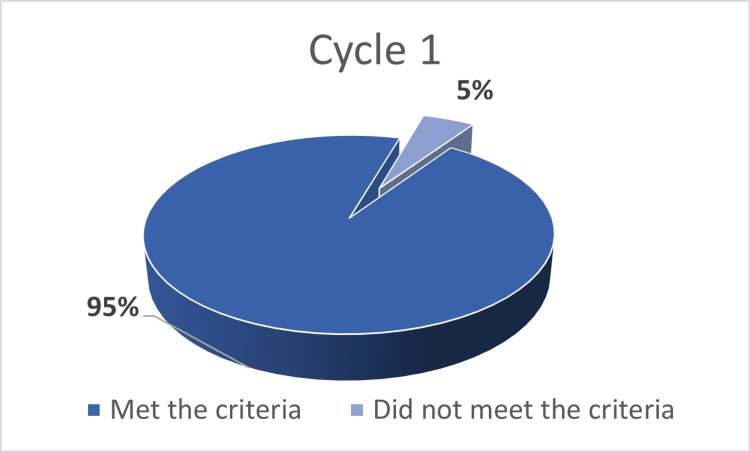
Results of the first cycle of the audit In the first cycle of the audit, 95 out of 100 CT scan reports (95%) met the RCR reporting standards, while five reports failed to meet the RCR standards for actionable reporting. CT: computed tomography; RCR: Royal College of Radiologists

**Figure 2:**
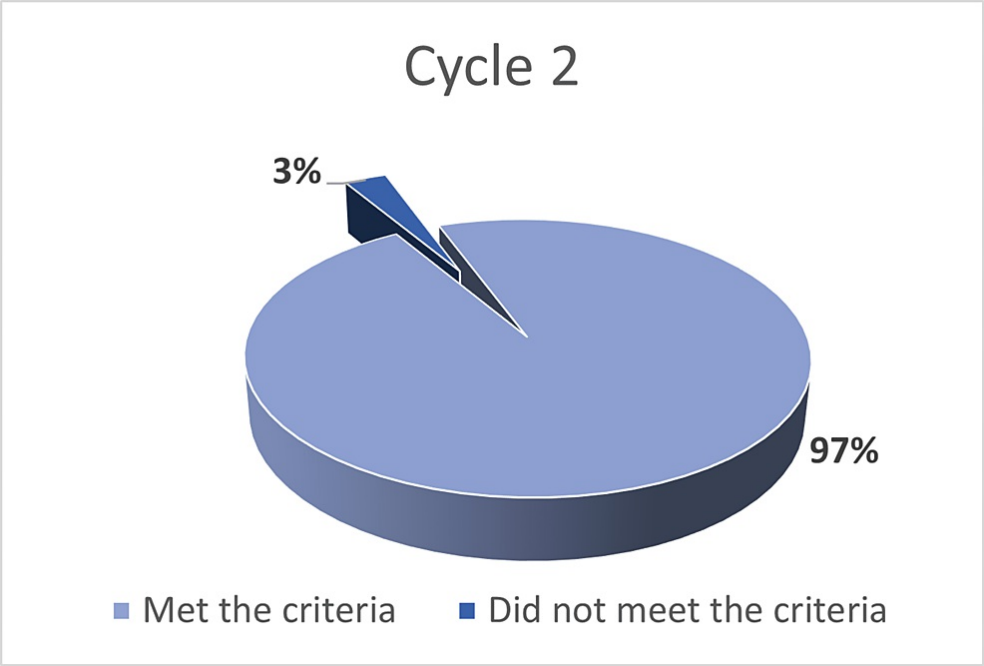
Results of the second cycle of the audit The second cycle of the audit assessed 100 randomly selected CT scan reports, with 97 reports (97%) meeting the RCR standards with three reports failing to meet the standards for actionable reporting. CT: computed tomography; RCR: Royal College of Radiologists

**Figure 3:**
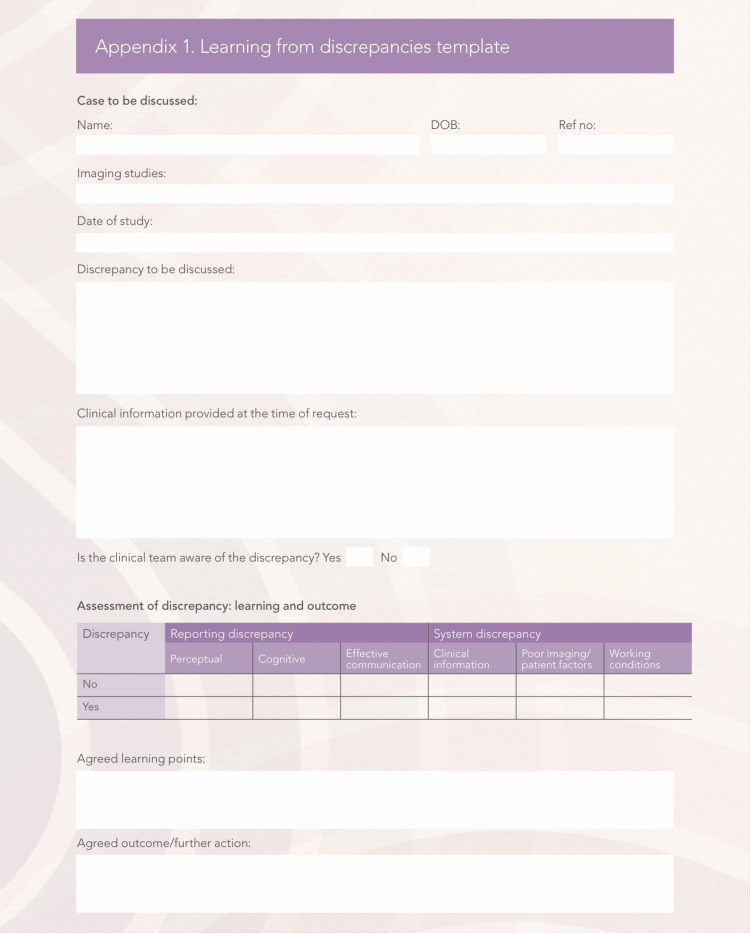
Royal College of Radiologists perform a used “Learning from discrepancies template” Template used to review all computed tomography (CT) scans and provide feedback to the radiologists reporting the scans.

In the first cycle of the audit, 95/100 (95%) CT scan reports met the RCR reporting standards, while five reports failed to meet these standards (Figure 1). This compared to 97/100 (97%) of reports meeting the RCR standards, with three reports, one from the inpatient database and two from the accident and emergency (A&E) department, failed to meet the standards (Figure 2). This did not reach statistical significance (p=0.397, Chi-squared test).

For the first cycle, 3/5 (60%) of the non-compliant reports originated from the inpatient database, while 2/5 (40%) were from the A&E department. The non-compliance was noted in the deficiency to advise on the next appropriate step of management in 4/5 (40%) of the reports, and failure to answer the clinical question in one report. The examples of reports that failed to meet the RCR standards in the first audit cycle are presented in (Table 2).

**Table 2:** Examples of reports not meeting the RCR standards in the first audit cycle The table demonstrates the examples of non-actionable reporting, identified in the first audit cycle in August 2021. CT: computed tomography; RCR: Royal College of Radiologists; IR: interventional radiology; UTI: urinary tract infection; NG tube: nasogastric tube; SPR: specialist registrar; I/P: inpatient; A&E: accident and emergency

Imaging Request	Clinical Question	Report and Errors Detected
I/P CT Whole Body	Follow up of psoas abscess after IR drainage	Mentioned comments on the change in psoas abscess but didn’t mention regarding feasibility or need for repeat drainage
I/P CT Whole Body	To identify the possible source of sepsis	Large hepatic abscess but no advice on management regarding drainage was given
A&E CT Abdomen and pelvis with contrast	Known carcinoma ovary with an incisional hernia and with complaints of abdominal pain and vomiting	Mentioned obstructed hernia and perforation but didn’t mention surgical referral and +/- NG tube insertion (reported by on-call SPR and agreed by on-call consultant)
A&E CT Abdomen and pelvis with contrast	To rule out obstruction	Mentioned large bowel obstruction secondary to CA sigmoid colon but didn’t mention urgent surgical referral and colorectal MDT (reported by on-call SPR and agreed by on-call consultant)
I/P CT Urinary Tract with contrast	To identify the cause of urosepsis and recurrent UTIs	Mentioned improved appearances from the last scan but didn’t answer the clinical question of the potential cause of infection

In response to the findings of the first audit cycle, a departmental presentation was made to emphasize the importance of reporting according to the RCR guidelines. The presentation was also circulated to individuals who were unable to attend in person.

The non-compliance in these reports was noted as a lack of specification of the next step of management, as the reports only described the collections but did not indicate the feasibility or non-feasibility of percutaneous drainage. The examples of reports that failed to meet the RCR standards in the second audit cycle are presented in (Table 3).

**Table 3:** Examples of reports not meeting the RCR standards in the second audit cycle The table demonstrates the examples of non-actionable reporting, identified in the second audit cycle in October 2021. RCR: Royal College of Radiologists; CT: computed tomography; I/P: inpatient; A&E: accident and emergency

CT Scan Performed	Clinical Question and Errors Detected
CT Abdomen and pelvis with contrast (I/P and A&E)	Regarding collections. However, there was no clear indication regarding feasibility/non-feasibility for percutaneous drainage mentioned in the report.

## Discussion

Diagnostic imaging is a medical act that is essential to all patient care and interventions, and radiologists carry clinical responsibility for that imaging by providing a medical opinion. Radiologists receive training to ensure competency and consistency across all imaging systems and clinical scenarios. The interpretation and reporting of imaging investigations are dependent on broader clinical and professional interactions, in which working in teams, governed by governance structures that review individual work, delivers great benefit for patient care [3,4]. The RCR standards for actionable reporting detail what patients should expect and emphasize the importance of actionable reporting, teamworking, close communication, peer feedback, and learning and system improvement [4].

The use of CT scans in the diagnostic process of trauma patients and critically unwell patients with multiple is vital for the patient outcome. In addition to clinical assessments, CT scans play a significant role in diagnosing these patients and improving the accuracy of the diagnosis. However, it is crucial that the reports generated from these scans are of high quality and meet the necessary standards in order to ensure their accuracy and reliability alongside prompt diagnosis in the management of patients in hospitals. CT scans play a vital role in the diagnosis of patients, in addition to clinical assessments. The reporting of CT scans should adhere to the standards set by the RCR in order to ensure the quality of the diagnostic process [3,5-7].

The use of structured reporting templates and checklists in the reporting process can significantly improve the accuracy and completeness of CT scan reports [4]. Templates provide a standardized format for the reporting process, which ensures that all important information is included in the report, while checklists provide a systematic approach to the reporting process, reducing the risk of missing important information. The use of online portals, such as CRIS, provides a centralized platform for the reporting process, ensuring that all reports are consistent and meet the necessary standards, improving the efficiency and accuracy of the reporting process [8,9].

The prompt diagnosis and reporting of CT scans are vital components in the management of critically injured patients with multiple traumas. The use of CT scans in the diagnostic process can significantly improve patient outcomes, and the use of structured reporting templates, checklists, and online portals can ensure that the reports generated from these scans are accurate, reliable, and meet the necessary standards [10-12].

However, we noted that the limitations of our study were that it was a single-center study. Secondly, since this was only conducted at a Level 1 Major Trauma Center, it would be helpful to know what reporting standards are like at other hospitals in the United Kingdom. To overcome this we would recommend future research could focus on a collaborative study of United Kingdom hospitals to audit correspondence with reporting of RCR studies as there is a deficiency of empirical data in the research community.

It is essential for healthcare institutions to regularly assess the quality of their CT scan reporting process through quality improvement projects and take necessary measures to improve the accuracy and reliability of their reports. The aim of any radiological imaging and reporting system is to provide prompt, actionable information [13]. To aid this the use of online portals, such as CRIS, can also help to improve the quality of CT scan reports. The portals provide a centralized platform for the reporting process, which can help to ensure that all reports are consistent and meet the necessary standards. The use of online portals can improve the efficiency of the reporting process, as well as the accuracy and reliability of the reports. Delays in reporting have an overall cost impact on the NHS (National Health Service) through delay in appropriate patient care and management and issues arising from this and patient safety and optimal outcomes needs prompt actionable reporting of actionable investigation [13-15].

## Conclusions

In conclusion, the use of RCR standards in the reporting process of CT scans can significantly improve the quality of the diagnostic process. The results of this study show that almost 97% of reports met the standards, compared to 95% in the first cycle. It is important to continue to evaluate the reporting process and make necessary improvements to ensure that all reports meet the necessary standards. This can improve patient outcomes by reducing the risk of diagnostic errors and ensuring that all necessary information is included in the reports.
